# Supplementary figures and images for: Molecular probes of spike ectodomain and its subdomains for SARS-CoV-2 variants, Alpha through Omicron
Source: PLoS One. 2022 May 24;17(5):e0268767. doi: 10.1371/journal.pone.0268767 (PMC9129042; doi:10.1371/journal.pone.0268767)

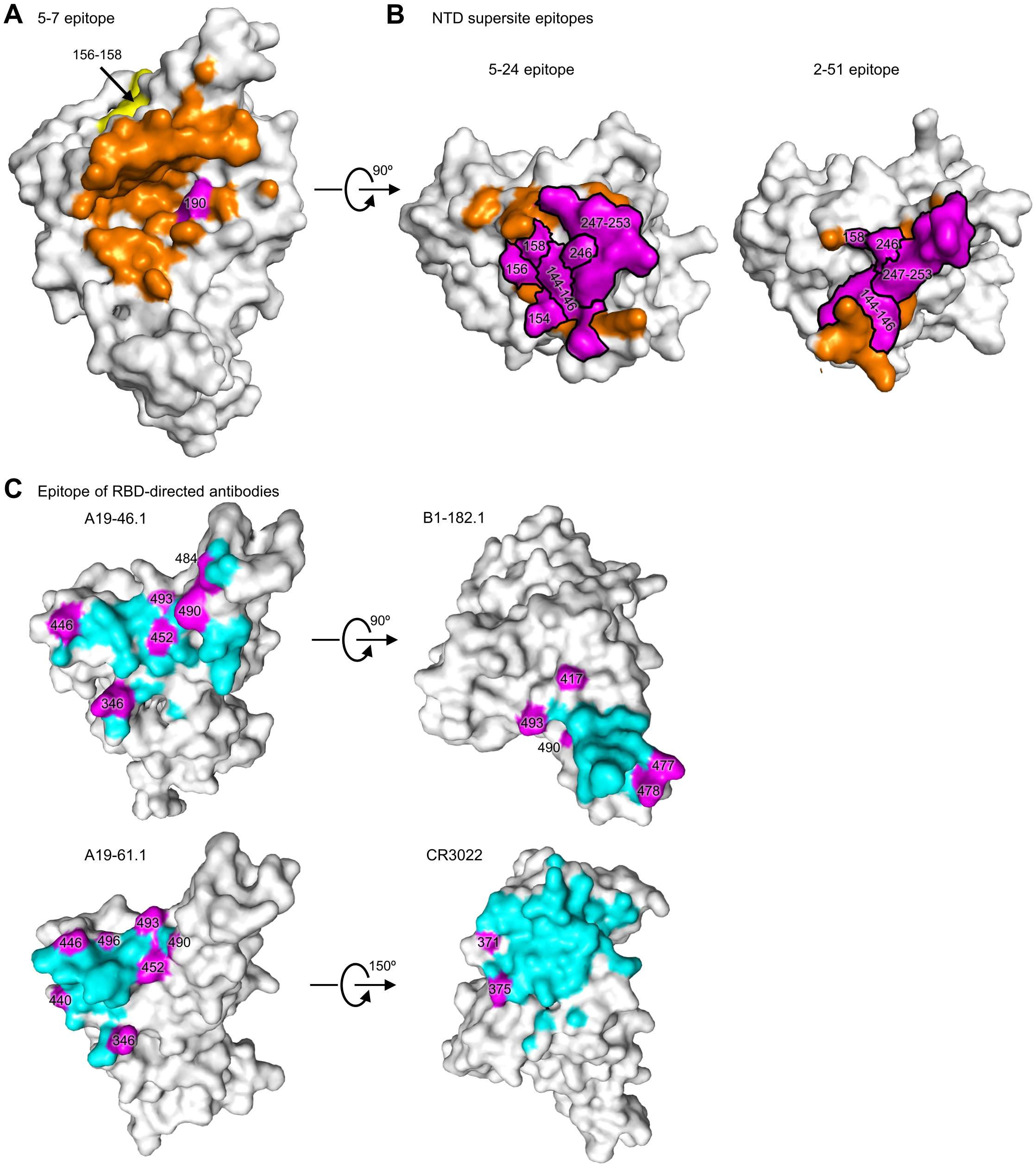

Supplement: S1 Fig — Epitopes of NTD-directed antibodies are colored in orange and those of RBD-directed antibodies in cyan, with variant mutations inside the epitopes highlighted in magenta and residue numbers labeled. (A) NTD antibody 5–7 (PDB: 7RW2). Residues 156–158 that are outside 5–7 epitope but likely to affect the epitope structure are highlighted in yellow. (B) NTD antibodies 5–24 (PDB: 7L2F) and 2–51 (PDB: 7L2C) that target the NTD supersite. (C) RBD-directed antibodies A19-46.1 (PDB: 7U0D), A19-61.1 (PDB: 7TBF), B1-182.1 (PDB: 7TBF), and CR3022 (7LOP). (TIF) [file pone.0268767.s001.tif]

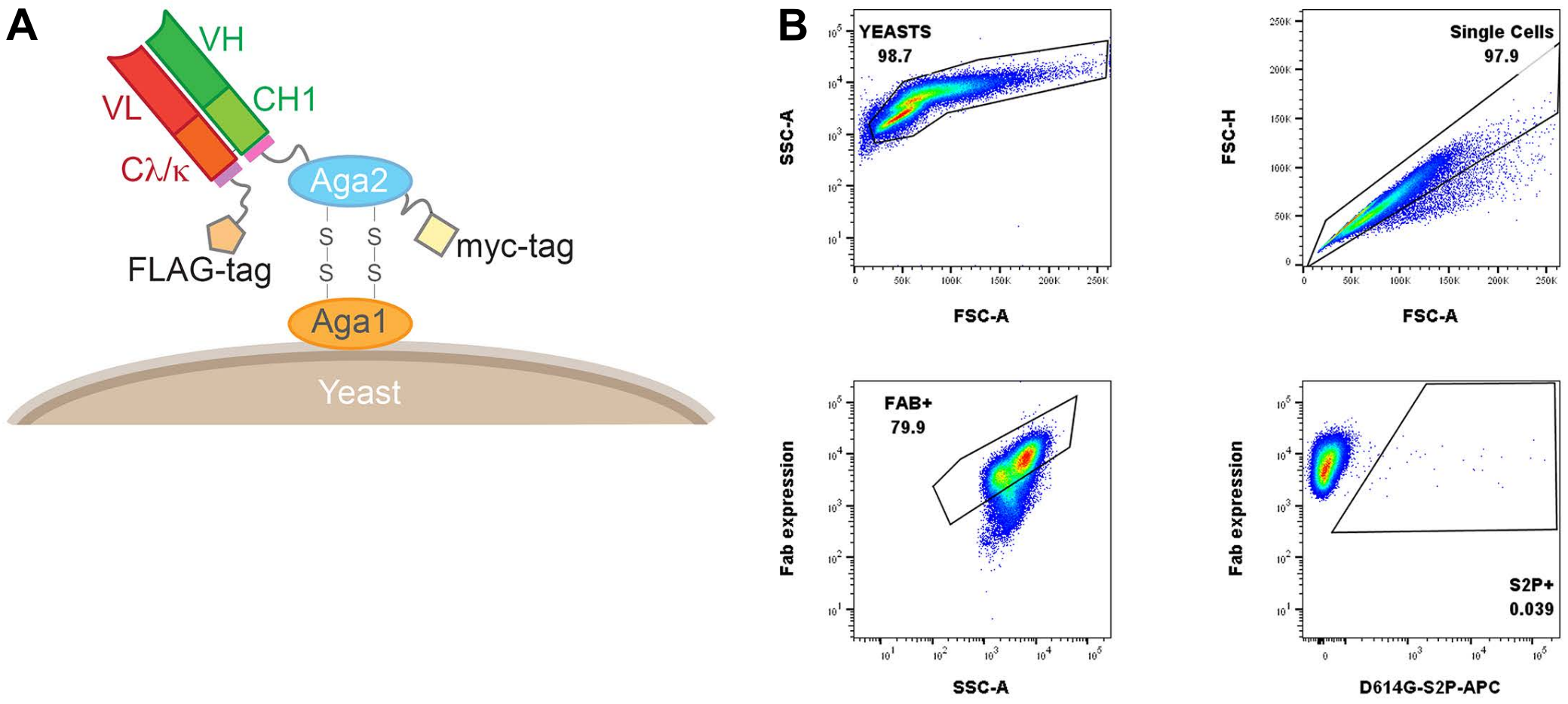

Supplement: S2 Fig — (A) Saccharomyces cerevisiae strain AWY101 transfected with yeast display vector and Fab display is induced by incubating yeast in galactose containing media. The presence of Fab expressed on the yeast surface can be detected by staining with an anti-Flag antibody and analyzing using flow cytometry. (B) Induced yeast bearing Fabs of interested are analyzed by the indicated gating strategy. Singlets are analyzed for Fab expression and the proportion of probe binding determined within this population of yeast. Shown is a representative data. (TIF) [file pone.0268767.s002.tif]

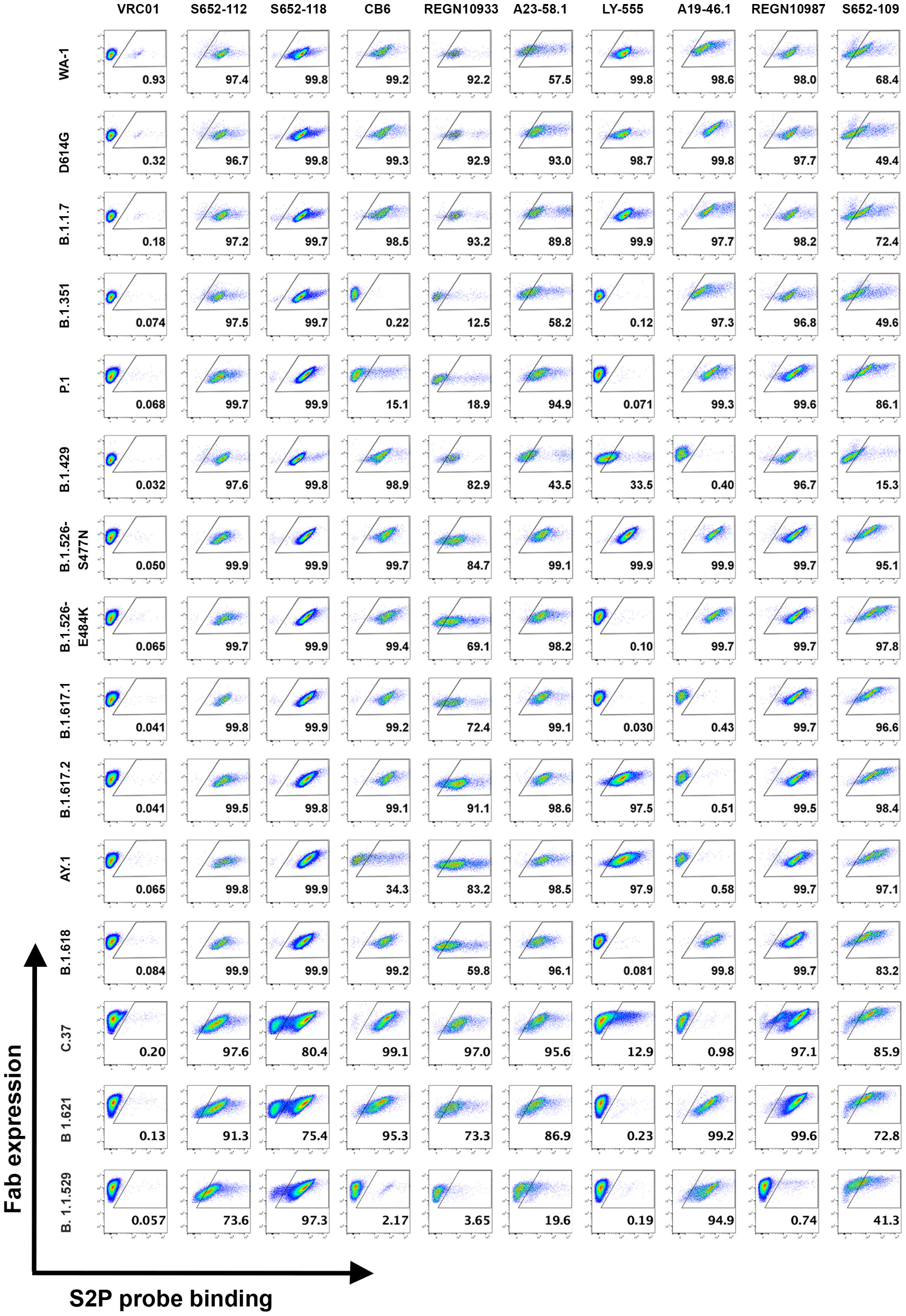

Supplement: S3 Fig — Binding of yeast expressing SARS-CoV cross-reactive Fabs (S652-118, S652-112, and S652-109), SARS-CoV-2 Fabs (LY-555, CB6, REGN10933, REGN10987, A19-46.1, and A23-58.1) or HIV targeting VRC01 Fab to SARS-CoV-2 VOC, VOI and other variant antigenic probes: WA-1, D614G, B.1.1.7, B.1.351, P.1, B.1.429, B.1.526-S477N, B.1.526-E484K, B.1.617.1, B.1.617.2, AY.1, and B.1.618 S2P (APC). (TIF) [file pone.0268767.s003.tif]

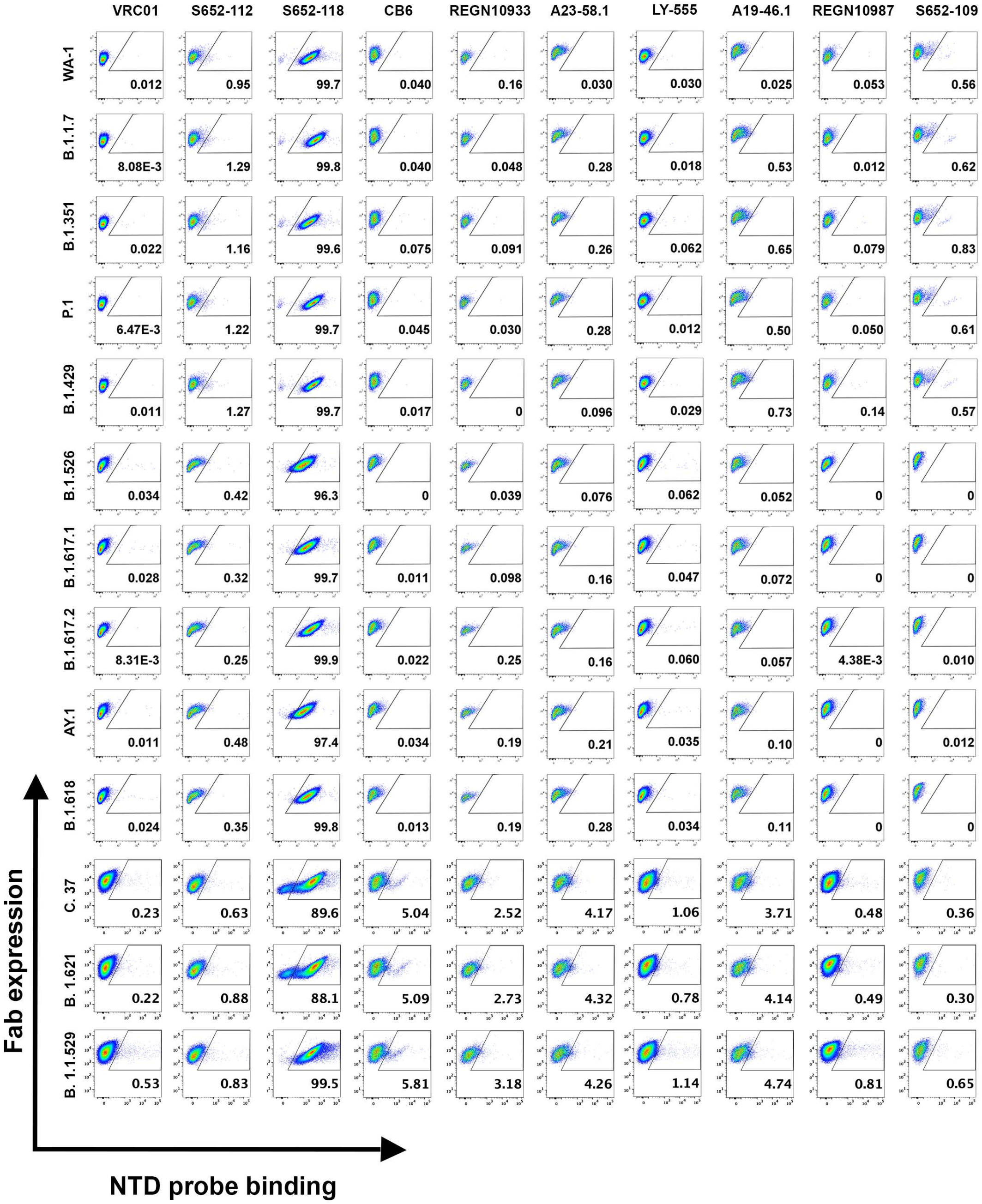

Supplement: S4 Fig — Binding of yeast expressing SARS-CoV cross-reactive Fabs (S652-118, S652-112, and S652-109), SARS-CoV-2 Fabs (LY-555, CB6, REGN10933, REGN10987, A19-46.1, and A23-58.1) or HIV targeting VRC01 Fab to SARS-CoV-2 VOC, VOI and other variant antigenic probes: WA-1, B.1.1.7, B.1.351, P.1, B.1.429, B.1.526, B.1.617.1, B.1.617.2, AY.1, and B.1.618 NTD (BV711). (TIF) [file pone.0268767.s004.tif]

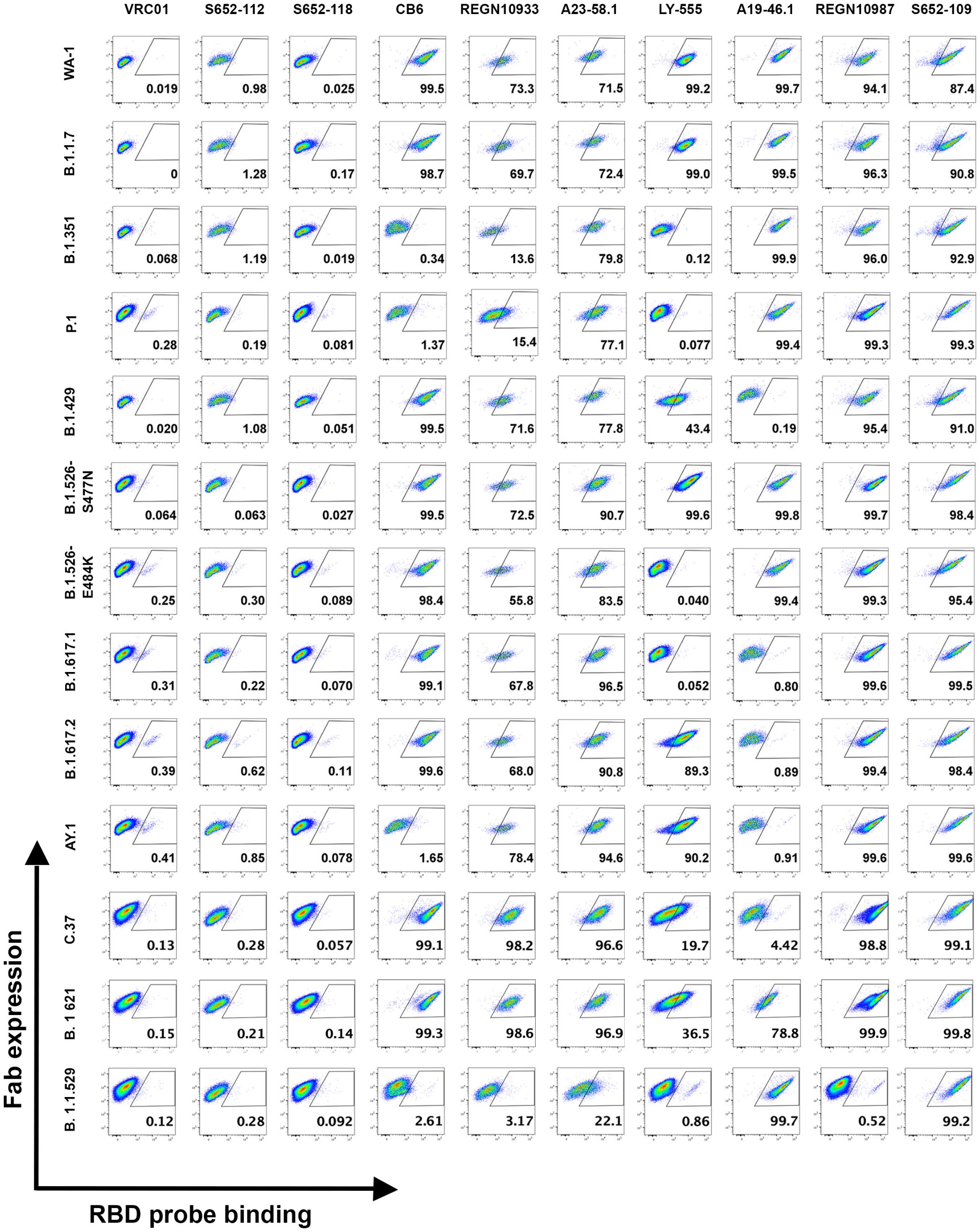

Supplement: S5 Fig — Binding of yeast expressing SARS-CoV cross-reactive Fabs (S652-118, S652-112, and S652-109), SARS-CoV-2 Fabs (LY-555, CB6, REGN10933, REGN10987, A19-46.1, and A23-58.1) or HIV targeting VRC01 Fab to SARS-CoV-2 VOC, VOI and other variant antigenic probes: WA-1, B.1.1.7, B.1.351, P.1, B.1.429, B.1.526-S477N, B.1.526-E484K, B.1.617.1, B.1.617.2, and AY.1 RBD (BV421). (TIF) [file pone.0268767.s005.tif]

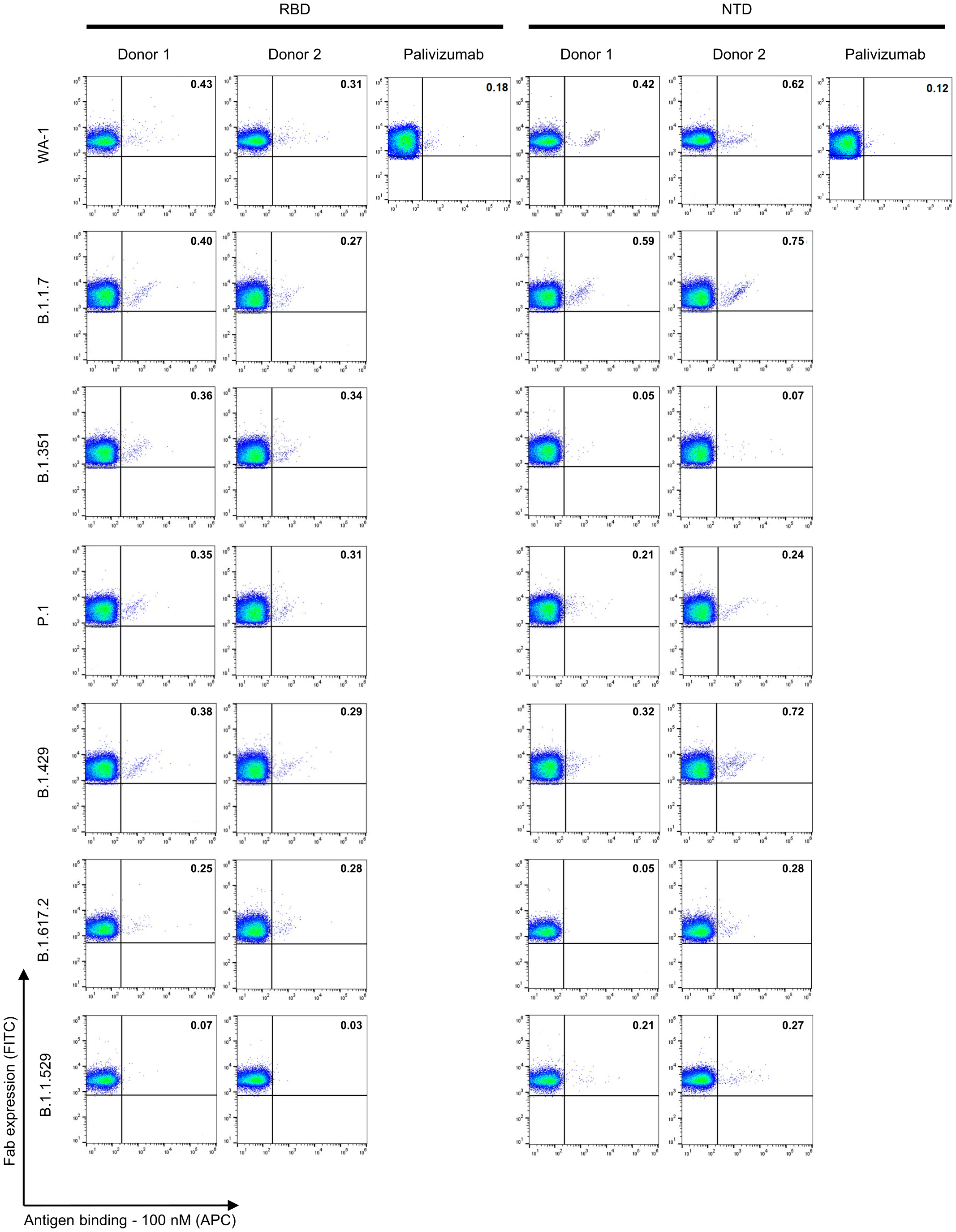

Supplement: S6 Fig — Binding of yeast expressing SARS-CoV-2 libraries (donor 1 and donor 2), targeting RBD and NTD of SARS-CoV-2 variants: B.1.1.7, B.1.351, P.1, B.1.429, and B.1.617.2. (TIF) [file pone.0268767.s006.tif]

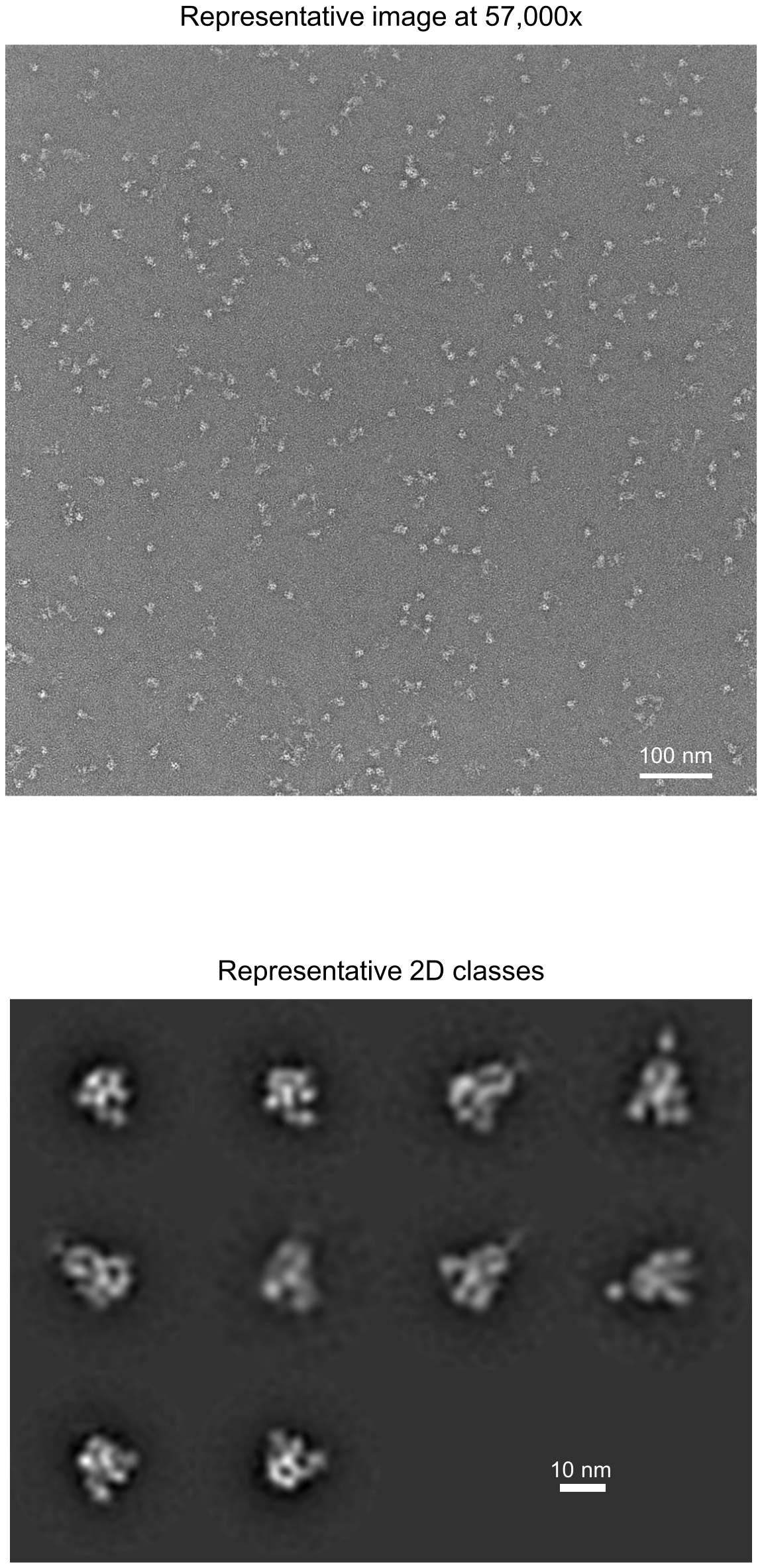

Supplement: S7 Fig — The top panel is the representative micrograph; the bottom panel shows the 2D-class averages. Sizes of scale bars are as indicated. At pH 5.5, B.1.1.529 S2P probe showed mostly trimeric particles with shapes similar to other S2P probes. (TIF) [file pone.0268767.s007.tif]
