# Supplementary material for: Molecular probes of spike ectodomain and its subdomains for SARS-CoV-2 variants, Alpha through Omicron
Source: PLoS One. 2022 May 24;17(5):e0268767. doi: 10.1371/journal.pone.0268767 (PMC9129042; doi:10.1371/journal.pone.0268767)
Supplement: S1 Table — (PDF) [file pone.0268767.s008.pdf]

S1 Table. Plasmids from this study and their Addgene accession numbers.

Plasmid name	Addgene #
pVRC8400-SARS-CoV-2-S2P-AVI	160474
pVRC8400-SARS-CoV-2-S2P-D614G-AVI	176324
pVRC8400-SARS-CoV-2-S2P-B.1.1.7-AVI	176325
pVRC8400-SARS-CoV-2-S2P-B.1.351-AVI	176326
pVRC8400-SARS-CoV-2-S2P-P.1-AVI	176327
pVRC8400-SARS-CoV-2-S2P-B.1.429-AVI	176328
pVRC8400-SARS-CoV-2-S2P-B.1.526-S477N-AVI	176329
pVRC8400-SARS-CoV-2-S2P-B.1.526-E484K-AVI	176330
pVRC8400-SARS-CoV-2-S2P-B.1.617-E154K-L452R-E484Q-D614G-P681R-AVI	176331
pVRC8400-SARS-CoV-2-S2P-B.1.617-G142D-E154K-L452R-E484Q-D614G-P681R-Q1071H-H1101D-AVI	176332
pVRC8400-SARS-CoV-2-S2P-B.1.617.1-AVI	176333
pVRC8400-SARS-CoV-2-S2P-B.1.617.2-AVI	176334
pVRC8400-SARS-CoV-2-S2P-AY.1-AVI	176335
pVRC8400-SARS-CoV-2-S2P-B.1.618-AVI	176336
pVRC8400-SARS-CoV-2-S2P-C.37-AVI	181693
pVRC8400-SARS-CoV-2-S2P-B.1.621-AVI	181694
pVRC8400-SARS-CoV-2-S2P-B.1.1.529-AVI	181695
pVRC8400-SARS-CoV-2-NTD-AVI	160475
pVRC8400-SARS-CoV-2-NTD-B.1.1.7-AVI	176424
pVRC8400-SARS-CoV-2-NTD-B.1.351-AVI	176426
pVRC8400-SARS-CoV-2-NTD-P.1-AVI	176429
pVRC8400-SARS-CoV-2-NTD-W152C-AVI	176431
pVRC8400-SARS-CoV-2-NTD-T95I-D253G-AVI	176432
pVRC8400-SARS-CoV-2-NTD-E154K-AVI	176433
pVRC8400-SARS-CoV-2-NTD-G142D-E154K-AVI	176434
pVRC8400-SARS-CoV-2-NTD-B.1.617.1-AVI	176435
pVRC8400-SARS-CoV-2-NTD-B.1.617.2-AVI	176436
pVRC8400-SARS-CoV-2-NTD-AY.1-AVI	176437
pVRC8400-SARS-CoV-2-NTD-B.1.618-AVI	176438
pVRC8400-SARS-CoV-2-NTD-C.37-AVI	181696
pVRC8400-SARS-CoV-2-NTD-B.1.621-AVI	181697
pVRC8400-SARS-CoV-2-NTD-B.1.1.529-AVI	181698
pVRC8400-SARS-CoV-2-RBD-AVI	160476
pVRC8400-SARS-CoV-2-RBD-N501Y-AVI	176439
pVRC8400-SARS-CoV-2-RBD-K417N-E484K-N501Y-AVI	176440
pVRC8400-SARS-CoV-2-RBD-K417T-E484K-N501Y-AVI	176441
pVRC8400-SARS-CoV-2-RBD-L452R-AVI	176442
pVRC8400-SARS-CoV-2-RBD-S477N-AVI	176443
pVRC8400-SARS-CoV-2-RBD-E484K-AVI	176444
pVRC8400-SARS-CoV-2-RBD-L452R-E484Q-AVI	176445
pVRC8400-SARS-CoV-2-RBD-L452R-T478K-AVI	176446
pVRC8400-SARS-CoV-2-RBD-K417N-L452R-T478K-AVI	176447
pVRC8400-SARS-CoV-2-RBD-C.37-AVI	181699
pVRC8400-SARS-CoV-2-RBD-B.1.621-AVI	181700
pVRC8400-SARS-CoV-2-RBD(aa335-526)-B.1.1.529-AVI	181701
pVRC8400-SARS-CoV-2-RBD(aa330-526)-B.1.1.529-AVI	182930
pVRC8400-SARS-CoV-2-RBD-SD1-WA1-AVI	176448
pVRC8400-SARS-CoV-2-RBD-SD1-N501Y-A570D-AVI	176449
pVRC8400-SARS-CoV-2-RBD-SD1-K417N-E484K-N501Y+A76-AVI	176450
pVRC8400-SARS-CoV-2-RBD-SD1-K417T-E484K-N501Y-AVI	176451
pVRC8400-SARS-CoV-2-RBD-SD1-L452R-AVI	176452
pVRC8400-SARS-CoV-2-RBD-SD1-S477N-AVI	176453
pVRC8400-SARS-CoV-2-RBD-SD1-E484K-AVI	176454
pVRC8400-SARS-CoV-2-RBD-SD1-L452R-E484Q-AVI	176455
pVRC8400-SARS-CoV-2-RBD-SD1-L452R-T478K-AVI	176456
pVRC8400-SARS-CoV-2-RBD-SD1-K417N-L452R-T478K-AVI	176457
pVRC8400-SARS-CoV-2-RBD-SD1-C.37-AVI	181702
pVRC8400-SARS-CoV-2-RBD-SD1-B.1.621-AVI	181703
pVRC8400-SARS-CoV-2-RBD-SD1-B.1.1.529-AVI	181704
