# Supplementary material for: Molecular probes of spike ectodomain and its subdomains for SARS-CoV-2 variants, Alpha through Omicron
Source: PLoS One. 2022 May 24;17(5):e0268767. doi: 10.1371/journal.pone.0268767 (PMC9129042; doi:10.1371/journal.pone.0268767)
Supplement: S2 Table — (PDF) [file pone.0268767.s009.pdf]

S2 Table. Plasmids for BA.2 variant and their Addgene accession numbers.

Plasmid name	Addgene #
pVRC8400-SARS-CoV-2-S2P-BA.2-AVI	184531
pVRC8400-SARS-CoV-2-NTD-BA.2-AVI	184532
pVRC8400-SARS-CoV-2-RBD-BA.2-AVI	184533
pVRC8400-SARS-CoV-2-RBD-SD1-BA.2-AVI	184534
